# The Influence of Organic Acids upon the Salivary Conversion of Starch

**Published:** 1891-04

**Authors:** 


### The Influence of Organic Acids upon the Salivary Conversion of Starch.

Salkowski investigated in the course of his trials regarding the usefulness of saccharine, the digestion of starch under the influence of this agent. He discovered that a concentrated and even a five-times diluted solution of saccharine entirely inhibits the influence of the salivary ferment upon starch. He regards this effect exclusively as an acid effect, because it entirely disappears when the mixtures are neutralized. John investigated the influence of a large number of organic acids upon the starch conversion, and found that the organic acids of the fat series in very small proportion (hydrochloric acid also) promote the saccharification of starches by mixed unfiltered saliva of alkaline reaction. This effect depends upon a union with the acids. By means of small quantities of free acids an inhibition of salivary action is induced, the inhibition coefficient stands in no relation to the chemical constitution of the acid. Oxalic acid, which acts most poisonously upon the general system, also possesses the greatest power of inhibition upon the diastatic process; acetic acid, which is most commonly used, possesses almost the least, a fact which probably may be explained upon the theory of adaptation. It is remarkable, too, that the apparently innocent tartaric acid exercises a very powerful inhibitory influence. Hence, it would appear that a union of the salivary ferment with an acid is not the effective principle of the inhibition. This effect of organic acid upon the salivary enzyme may be compared, on account of its want of amenability to any law, to the effect of an anti-bacterial agent upon the vital process of the lowest life elements. *Virchow's Archiv.*

---

INFANTILE CONVULSIONS.—Dr. Jacobi first orders a purgative dose of calomel and then follows in a few hours by:

R. Chloral hydrat........................ iv grains.
Potas. bromid............................ viij grains.
Aquæ }
Syrupi } .................................. aa j fluid drachm.

Sig. One dose for a child 2 years old.

THE INTERNATIONAL MEDICAL CONGRESS AT BERLIN.—The first volume of the *Transactions* of the International Medical Congress at Berlin is already in the press, and will be published in November. In addition to the business part of the proceedings, lists of delegates, members, etc., it will contain the report of the general meetings of the congress. The work of editing the *Transactions* is in the hands of a committee composed of Professors Virchow, von Bergmann, and Waldeyer. The expenses incurred by the city of Berlin in connection with the recent International Medical Congress amounts to some 80,000 marks, so that of the sum of 100,000 marks voted by the municipality, a balance of 20,000 marks (£1,000) remains in hand. The expenses included the cost of the *Festschrift* presented to members of the congress, the banquet in the Rathhaus, the exhibition, the sanitary and other inspections, and the electric illumination.—*Brit. Med. Journal.*

SOLUBILITY OF NEW MEDICINES.—The following table of solubilities of some new medicines may be useful to some of our readers:

| One part of | Water | Is sol. in Alcohol. | Ether. |
|---|---|---|---|
| Antifebrine | 200 | 10 | 10 |
| Antipyrin | 1 | 1 | 50 |
| Antithermin | slightly soluble. | slightly soluble. | slightly soluble. |
| Cocaine hydrochlor | 5 | 10 | |
| Iodol | 5000 | 3 | 1 |
| Paraldehyde | 10 | | |
| Pyrodine | 1 | 1 | |
| Quinoline tartar | 80 | 150 | |
| Resorcin | 1 | 1 | |
| Salol | | 5 | 5 |
| Thallin (sulphate) | 7 | 100 | |
| Thallin tartrate | 10 | | |
| Urethane | 1 | 0.6 | |

*Pharm. Record.*

